# Prescribing Syringes to People Who Inject Drugs: Advancing Harm Reduction in Primary Care

**DOI:** 10.1007/s11606-023-08183-7

**Published:** 2023-04-05

**Authors:** Avik Chatterjee, Maxwell Bannister, Lucas G. Hill, Corey S. Davis

**Affiliations:** 1 Boston Health Care for the Homeless Program, Boston, MA USA; 2 Boston Medical Center, Boston, MA USA; 3 Boston University School of Medicine, Boston, MA USA; 4 Augsburg University, Minneapolis, MN USA; 5 The University of Texas at Austin College of Pharmacy, Austin, TX USA; 6 Network for Public Health Law, Edina, MN USA; 7 NYU Grossman School of Medicine, New York City, NY USA

## Abstract

Access to new syringes can reduce the risk of HIV and hepatitis C transmission, skin and soft tissue infections, and infectious endocarditis for people who inject drugs (PWID). Syringe service programs (SSPs) and other harm reduction programs are a good source of syringes. However, they are sometimes not accessible due to limited hours, geographic barriers, and other factors. In this perspective, we argue that when PWID faces barriers to syringes physicians and other providers should prescribe, and pharmacists should dispense, syringes to decrease health risks associated with syringe re-use. This strategy is endorsed by professional organizations and is legally permissible in most states. Such prescribing has numerous benefits, including insurance coverage of the cost of syringes and the sense of legitimacy conveyed by a prescription. We discuss these benefits as well as the legality of prescribing and dispensing syringes and address practical considerations such as type of syringe, quantity, and relevant diagnostic codes, if required. In the face of an unprecedented overdose crisis with many associated health harms, we also make the case for advocacy to change state and federal laws to make access to prescribed syringes uniform, smooth, and universal as part of a suite of harm reduction efforts.

“But doc, the syringe program is closed for the night, and I can’t afford to pay for new needles from my friends. What am I supposed to do?”

I practice shelter-based medicine in Boston, and I provide care to many people who inject drugs (PWID). Some of these individuals have abstinence as a goal and I am fortunate to have the resources to assist them in obtaining life-saving medications for substance use disorder (SUD), as well as other supportive services. But for many others, discontinuation of drug use is not the immediate goal; rather, they are mainly seeking ways to reduce the risks associated with lack of access to sterile syringes and other injection supplies including skin and soft tissue infections, infective endocarditis, viral infections such as HIV or hepatitis C, and overdose.^1^

I am thankful to be in a state and city (Boston, MA) that has a robust network of harm reduction organizations including numerous syringe service programs (SSPs). At SSPs, PWID can obtain naloxone to reverse overdoses, supplies like sterile water and alcohol pads to help inject safely, and new needles and syringes to prevent infections in a supportive, stigma-free environment. Decades of evidence show that SSPs reduce the risk of hepatitis C and HIV transmission, increase access to SUD treatment, and prevent overdose deaths.^2^ However, these services are often not available when and where they’re needed, as my patient let me know.

“The syringe exchange closed at 4 and the outreach workers just ran out of syringes. Syringes cost $5 a pop out on the streets and I just can’t afford that.” I knew that while this patient wanted to reduce his risk of potential harm by using a new syringe, if he could not find one he would be forced to re-use a syringe to stave off withdrawal, potentially one that had been used by someone else. What were my options to help him use safely? I sent a message to some of my colleagues, and they reminded me that, while the SSP was closed, many pharmacies were still open. They suggested prescribing needles for him.

Even in locations such as Boston with a robust network of harm reduction services, pharmacies can be a vital source of harm reduction supplies.^3^ One analysis in Massachusetts and Rhode Island found that a single pharmacy chain sold approximately ten-fold more non-prescription syringes than were distributed by all SSPs in those states over the course of a year.^4^ Many PWID are too far from SSPs to be able to utilize their services,^5^ or are unable to access them during operating hours. Conversely, pharmacies typically have longer hours of operation with some open around the clock. They are also much more likely to be available in rural areas as 89% of Americans live within 5 miles of a pharmacy.^6^ Furthermore, since they sell a wide variety of items, entering a pharmacy does not “out” a customer as a PWID. Additionally, Black people may be less likely to access SSPs than White or Hispanic people,^7^ so offering additional means by which they can access new syringes is important to begin addressing longstanding racial inequities in addiction treatment.

So I decided to prescribe needles for this patient. Even though no prescription is required to access syringes in Massachusetts, regardless of the number prescribed, prescribing syringes to PWID can have advantages.^8^

The first and likely most important advantage is cost. One of the key benefits of SSPs is that they provide supplies without charge. Even where syringes are available at pharmacies, they are not free without a prescription. However, insurance generally covers prescribed syringes, removing or decreasing the cost barrier that my patient cited for needles in the secondary market. Prescriptions do not generally require a diagnosis, and given that needles might be prescribed for other conditions such as diabetes, insurance coverage is generally not an issue.^9^ In my experience, the state Medicaid program pays for prescribed needles regardless of diagnosis, without a co-pay, making prescribed syringes for patients like mine financially accessible, though prescribers should check with the pharmacy or patient’s insurance plan to confirm the cost of syringes to the patient. In jurisdictions where Medicaid or other insurance programs do not cover syringes or do not cover them without a diagnosis, we recommend advocating for them to do so. In the meantime, if an insurance program asks for a diagnosis code, the following can be utilized: Z20.6 (contact with and (suspected) exposure to HIV), Z20.2 (contact with and (suspected) exposure to infection with a predominantly sexual mode of transmission), or Z77.21 (contact with and (suspected) exposure to potentially hazardous body fluids).

The second is the legitimacy implied by a prescription. Although syringes are available without a prescription, pharmacists may be unwilling to sell non-prescription syringes to PWID because of stigma or fear of “enabling” drug use.^10,11^ While education has been shown to decrease negative pharmacist attitudes towards PWID,^12^ some pharmacists may still refuse to sell syringes to those individuals. Having a prescription may make it less likely that the pharmacist will refuse, as in our experience the mere fact of the prescription implies that the use is “legitimate.” The prescription is, in a sense, transferring some of the privileges of the prescriber to the patient. We acknowledge that stigma remains an issue among physicians and other prescribers, and may limit the adoption of the practice of prescribing syringes.^13^ However, training can be effective to reduce stigma toward people with OUD^14^ and harm reduction practices,^15^ and should be instituted broadly and early in health professions training.

Laws regarding SSPs, the possession of syringes for use in injecting illegal drugs, and pharmacist dispensing of syringes to PWID vary widely by state and can be confusing and difficult to reconcile (Fig. 1). In Massachusetts, for example, the prescribing, dispensing, and possession of syringes for illicit drug use are all legal. In contrast, Georgia law prohibits the possession of syringes for illicit drug use, and explicitly states that no “hypodermic needle or syringe shall be sold by a pharmacist or pharmacy intern/extern…if such person has reasonable cause to believe that it will be used for an unlawful purpose.”^16^

**Figure 1:**
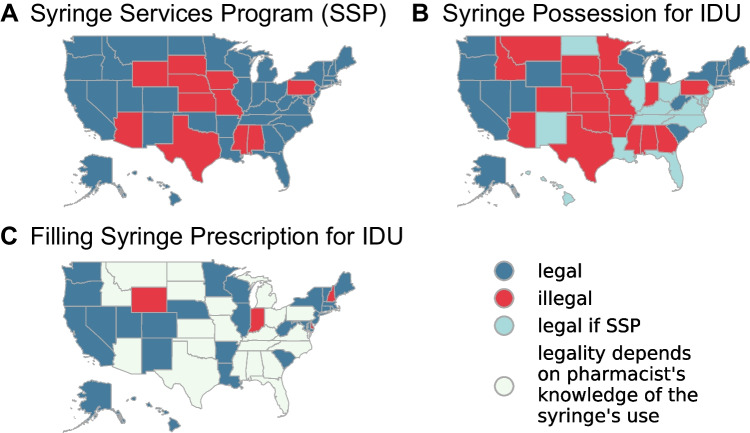
State-level legality of syringe service programs, possession of needles for the purpose of injection drug use (IDU), and filling a syringe prescription for IDU.

All states permit syringes to be accessed via a pharmacy without a prescription, although a small number require a prescription for syringes distributed to minors or in amounts greater than ten.^17^ Similarly, the prescribing of syringes to PWID does not generally violate state laws. A 2001 legal review found that such prescribing was prohibited in only two states (Delaware and Kansas),^18^ and the relevant language in the Delaware law has since been removed.

Of note, prescribing syringes does not make possession of syringes for use with illicit drugs legal if paraphernalia or other laws make their possession or distribution illegal. In many states, whether pharmacy dispensing of syringes is legal depends, at least from a technical legal perspective, on whether the pharmacist knows or should know whether the syringe will be used for illegal drugs. This is because many state paraphernalia laws forbid the distribution of devices such as syringes when the distributor knows or should know that they will be used to inject drugs in violation of state law. This is unfortunate, as it may encourage some pharmacists not to inquire about the patient’s intended use of the syringe. While this might theoretically reduce criminal legal risk to the pharmacist, it removes a potentially important opportunity for the pharmacist to offer other risk reduction tools such as education on safe injection techniques, fentanyl test strips, naloxone, and referral to SSP or SUD treatment resources.

In practice, however, the criminal legal risk to both prescribers who issue prescriptions for syringes to PWID and pharmacists who fill them is extremely low. We systematically searched Medline and the Nexis news archive for any instances of a physician or pharmacist being arrested for prescribing syringes to PWID or any outcomes related to that prescribing. We found no reports of such arrests, and research has found that civil liability risk related to prescribing syringes is extremely low.^19^ However, prescribers may wish to consult with a local attorney with questions regarding the legality of prescribing syringes in the state in which they practice.

Because syringes do not generally require a prescription, most recent scholarship has focused on the availability of nonprescription syringes from pharmacies.^20,21^ Recent results from a nationally representative sample show that obtaining syringes from a pharmacy is associated with reductions in syringe sharing and reuse compared to people who obtained syringes on the street.^22^ While the American Pharmacists Association does not currently have a stated policy on syringe prescribing to PWID, it is notable that they do have multiple policies supporting pharmacist dispensing of non-prescription syringes—and provision of other vital harm reduction supplies and education—to this population.^23^

Prescribing syringes is logistically very easy, and any primary care provider in any setting should feel comfortable doing so. A small number of physicians have successfully been prescribing syringes for harm reduction purposes for decades—in a 2000 survey of addiction medicine providers, 2% had prescribed syringes for use by PWID^24^—though the practice is limited by stigma, lack of knowledge, and confusing and unsupportive laws. Additionally, the practice has been endorsed by the American Public Health Association^25^ among other professional associations, for over twenty years.

Speaking logistically, if the patient does not raise the issue of safer injection practices themselves as this patient did, beginning the conversation by screening for substance use, method, and frequency of use would be a good place to start. Syringes are not controlled, so prescriptions for them can be written using paper prescription pads in most states; clinic-based practices can also create macros to enable them to be easily e-prescribed. In many cases, the patient will know the size of the syringe they typically use. If not, it is generally sufficient to write for a common size, such as 28 gauge, ½ inch, 1cc, (which my patients refer to as “Biggie Smalls”), and note that the pharmacist may substitute another size based on patient preference and availability. For quantity, we recommend having a conversation with the patient to determine the number of syringes they use and to what extent they are able to access an SSP or other harm-reduction program for syringes. The prescription might provide a supplemental number of syringes or be the sole source, but it is important that the quantity not be a limiting factor for safe injection practices. The clinician should use their judgment when deciding whether to initiate such a prescription to a particular patient, although outside of rare outlying incidents such as a patient who expresses a desire to self-harm by injecting drugs, we think there are few reasons not to prescribe syringes to any PWID who cannot otherwise access a sufficient supply of sterile injection equipment. We note that state law regarding the ability of minors to consent to medical care, including prescribing, varies among states.^26^

In a very real sense, the fact that prescribing syringes is still a method for increasing access to them is a policy failure. In a system focused on reducing drug-related harm and not criminalizing people who use drugs, SSPs would be universally permitted and well-funded, paraphernalia possession and distribution would not be prohibited, and syringes would be freely available from pharmacies. It is incumbent on those of us who favor evidence-based, patient-focused, non-stigmatizing policy to continue to advocate for the repeal of laws and policies that reduce access to syringes and other lifesaving harm-reduction supplies. In the meantime, prescribing syringes can be a stopgap strategy to facilitate safer injection practices. As overdose deaths reach ever-higher numbers, every option to prevent drug-related harms and engage people in treatment will be vital. This will involve health care providers prescribing needles in jurisdictions where they can, and advocating for policy change in jurisdictions where they currently cannot.^27^

Note to the reader: While this perspective was co-authored by a physician, a medical student, a pharmacist, and a lawyer, the narrative is presented from the standpoint of the physician lead author.

## Data Availability

The data used for this perspective piece is publicly available on the internet, as indicated in the citations.
